# Faculty development for translational simulation: a qualitative study of current practice

**DOI:** 10.1186/s41077-023-00265-0

**Published:** 2023-11-02

**Authors:** Victoria Brazil, Eve Purdy, Alexander El Kheir, Rebecca A. Szabo

**Affiliations:** 1 Faculty of Health Sciences and Medicine, Bond University, Gold Coast, QLD Australia; 2 Emergency Department, Gold Coast Hospital and Health Service, Gold Coast, QLD Australia; 3 Melbourne Medical School, The University of Melbourne, Melbourne, VIC Australia

**Keywords:** Healthcare simulation, Translational simulation, Faculty development

## Abstract

**Background:**

Translational simulation is focused directly on healthcare quality, safety, and systems. Effective translational simulation design and delivery may require knowledge and skills in areas like quality improvement and safety science. How translational simulation programs support their faculty to learn these skills is unknown. We aimed to explore current faculty development practices within translational simulation programs, and the rationale for the approaches taken.

**Methods:**

We used a qualitative approach to explore faculty development in translational simulation programs. We conducted semi-structured interviews with representatives who have leadership and/or faculty development responsibilities in these programs and performed a thematic analysis of the data.

**Results:**

Sixteen interviews were conducted with translational simulation program leaders from nine countries. We identified three themes in our exploration of translational simulation faculty development practices: (1) diverse content, (2) ‘home-grown’, informal processes, and (3) the influence of organisational context. Collaboration beyond the historical boundaries of the healthcare simulation community was an enabler across themes.

**Conclusion:**

Leaders in translational simulation programs suggest a diverse array of knowledge and skills are important for translational simulation faculty and report a range of informal and formal approaches to the development of these skills. Many programs are early in the development of their approach to faculty development, and all are powerfully influenced by their context; the program aims, structure, and strategy.

**Supplementary Information:**

The online version contains supplementary material available at 10.1186/s41077-023-00265-0.

## Background

The simulation community has embraced translational simulation (also described as ‘*systems focused’/”QI”*), and attendant novel approaches to simulation design [1, 2], delivery [1, 3–6], debriefing [7–9], and institutional engagement [2, 10]. However, the adoption of these simulation strategies has not been matched by clear guidance for training the practitioners who deliver translational simulations. Practitioners may need additional knowledge and skills that build on those required for educationally focused simulations, drawing upon expertise from fields such as safety science, quality improvement, human factors, and change management. Despite a lack of published practice, we are anecdotally aware that translational simulation programs worldwide support their faculty in developing these skills. We sought to explore and describe these practices—the objectives, pedagogies, and methods—in how practitioners are prepared for the design and delivery of translational simulation activities.

Translational simulation aims to directly improve patient care and healthcare systems, through diagnosing safety and quality issues and delivering simulation-based interventions [11]. This approach “seeks to drive organizational learning by targeting simulation at the systems level as well as its components” [12]. Other terminologies and conceptual framings are offered for what we are referring to as ‘translational simulation’, including in situ simulation (ISS) [13], systems integration simulation [14], simulation-based clinical systems testing (SBCST) [15], and transformative (‘non-pedagogical’) simulation [16]. Common to these conceptualisations is (1) a direct focus on healthcare safety, quality, and systems, as distinct from simulation for pedagogical purpose, and (2) simulation design and delivery methods that draw upon healthcare improvement, design thinking, systems science and other fields of practice that may be unfamiliar to educators [1, 2, 15, 17].

Dedicated knowledge and skills are necessary for effective healthcare simulation delivery, irrespective of the purpose of the simulation activity. Widespread faculty development activities in the broader simulation community reflect this powerful belief. There is comprehensive guidance for practical design, delivery, and debriefing [18–24] of simulation-based education, and for developing simulation faculty development programs [25–29]. This published guidance is matched by a plethora of faculty development opportunities, including courses and workshops [30], formal simulation fellowship programs [31, 32], and online resources [33]. However, most of this faculty development occurs within an educational paradigm [25], i.e., faculty are described as simulation *educators*, simulation participants are *learners*, scenarios are written with individual or team *learning objectives*, debriefings are judged according to *transfer of learning*, and simulation faculty often have educational responsibilities for their participants. While this paradigm serves educationally focused simulation well, it may not be sufficient for translational simulation that is focused on *organisational* learning or *systems* improvement.

Effective faculty development for translational simulation will require consideration of both *what* needs to be learned, *how* those skills and perspectives are acquired, and *who* are the recipients of development activities. The first issue is content—what needs to be learned to design and deliver simulation that explores and improves healthcare system performance? Translational simulation practice may require a different strategic [11, 34] and operational [1] approach to educationally focused simulations. With limited published guidance to draw upon, we find diverse methods and tools—from quality improvement [35–37], safety science [9, 38], human factors/ergonomics [39, 40], design thinking [17], change management [2, 10], implementation science [41] and systems engineering [42]—have been used in translational simulation activities [1, 40]. These are often unfamiliar perspectives and skillsets for simulation practitioners. But established skill sets drawn from simulation-based education (SBE) remain necessary and foundational. These include debriefing, coaching, feedback, scenario design, educational theory and principles, evaluation of learning, curricular integration, operations, simulation research and scholarship, and simulation standards and theoretical support [25]. Adaptation and application of these foundational skills to translational contexts is an added nuance.

The second issue is method—how should the knowledge and skills to deliver translational simulation be learned? Literature on faculty development for health professions education [43, 44] and for healthcare simulation more broadly [25, 27] suggest a range of methods: learning from experience, learning from peers, mentorship, workshops seminars, and longitudinal programs, and work-based learning and communities of practice [45–48]. Faculty development pathways with graduated, or ‘tiered’ [27] approaches are common in well-established simulation programs, and credentialing [49] has supported consistency in the field. Foundational principles of instructional design for faculty development will apply equally to the translational simulation context, but how translational simulation programs select specific tools and approaches is likely to be context-dependent [46, 48]. This will depend on program aims, resources, constraints, existing skill sets, and whether there are experts qualified to conduct formal programs.

The third issue is identifying the practitioners—who receives this training, and who delivers it? Various descriptors are used for the personnel who design, deliver, and administer simulation programs more broadly: educators, coordinators, embedded participants, operations specialists, technicians, faculty, managers, and others. Cognisant that translational simulation may bring even more diversity to simulation programs—e.g., experts in human factors, quality improvement, and systems science—we have chosen the terminology ‘practitioner’ to describe those who may be recipients of faculty development activities within translational simulation programs.

With these three questions in mind, we aimed to explore current faculty development practices within translational simulation programs, and the rationale for the approaches taken. We were interested in issues of content and method, and the interrelationships between the two. With an informed picture of current faculty development strategies, our aspiration is that translational simulation programs can move toward greater consistency and effectiveness in their own efforts.

## Methods

We used a qualitative approach to explore faculty development in translational simulation programs across the globe. The Standards for Reporting Qualitative Research (SRQR) [50] guides our description of our methods. We collected data through semi-structured interviews with representatives of translational simulation programs who have leadership and/or faculty development responsibilities. We aimed to develop a picture of (1) the knowledge and skills considered important for translational simulation faculty, (2) the methods by which these knowledge and skills are acquired, and (3) reflections on the strengths, weaknesses, and opportunities in current approaches. The interview guide was designed based on these three aims. Taking a constructivist stance, we recognised that addressing these questions would require interpretation and constructing meaning from participant responses, and that responses were drawn from heterogenous contexts and variable interpretations of the role of faculty development in translational simulation. We were interested in exploring links between the context and aims of translational simulation programs and their approaches to faculty development. We were aware that we had a baseline perception prior to our study—that there would be reliance on traditional approaches to healthcare simulation faculty development, coupled with emerging approaches relevant to healthcare systems, quality improvement, and safety perspectives.

### Recruitment

We used several strategies to identify a cross-section of key informants from translational simulation leadership and faculty development. We took a purposive sampling approach and included (1) authors of published articles on translational simulation or ‘systems-focused’ simulation, (2) leaders in translational simulation program, as identified through professional networks, (3) attendees at a translational simulation workshop at the Society for Simulation in Europe (SESAM) conference in Seville, Spain, June 2022 who had consented to future contact for this purpose. We also used snowball sampling; recruited participants were asked if they had suggestions for additional translational simulation faculty development program leads whose perspectives’ they thought would be helpful for our work.

Our aim was to enhance understanding of this nascent field, not find absolute truth. As such, we sought a deep examination of the topic with a diverse group of participants. We felt this would provide a more comprehensive understanding than a widely distributed but more superficial survey approach. We recognize that our findings may not be representative of all translational simulation programs. We intended to sample from at least 15 programs from across the globe in the first instance, and then reassess the opportunities or need for further data collection. We contacted potential participants through email and invited them to participate in interviews. Additional file 1. Participant Information and Consent Form.

### The research team

VB is an emergency physician with 20 years of experience in healthcare simulation as a practitioner and researcher. She is the medical director of a translational simulation program in a large healthcare institution in Queensland, Australia, and author of articles about translational simulation. RS is an obstetrician gynaecologist and medical educator, with extensive experience in healthcare simulation, and who leads a translational simulation program in a women’s and newborn hospital in Melbourne Australia. She is undertaking a PhD in normalising translational simulation in teaching hospitals using qualitative methodology. AE is an emergency physician who has recently completed a fellowship in translational simulation with VB and with experience in the delivery of simulation faculty development workshops. EP is an emergency physician applied anthropologist, whose medical training was in Canada. She is an experienced qualitative researcher, including a healthcare simulation scholarship. She completed a translational simulation fellowship at the same institution where VB works. Overall, we felt that the benefits of proximity to the topic of translational simulation (allowing for granularity of data collection, rapport with participants, lived experience, and practical understanding) outweighed the potential risks. Throughout data collection and analysis this group reflected together on how their own positioning impacted data collection and interpretation and actively worked to identify opposing or new views.

### Data collection

Interviews were conducted by VB, RS, and AE between September and December 2022, using videoconferencing, and were 30–60 min in length. Interviews were audio recorded on the Zoom platform (Zoom Video Communications Inc. (San Jose, CA, USA, then transcribed using a cloud-based transcription service Otter.ai (Mountain View, CA, USA) (https://otter.ai/), followed by the interviewers checking and de-identifying the transcripts. The de-identified transcripts were sent to the study participants for checking and then analysed. There were no withdrawals from study participation after recruitment.

The interview guide is provided in Additional file 2. After the first 5 interviews, we met to discuss our initial impressions of the data and to reflect on the adequacy of the interview guide. At this stage, we added one question—“*Tell me about the experience of a new person who arrives and wants to do ‘translational sim’?*”—as we had found it to be a useful prompt in the initial interviews.

### Data analysis

We performed an inductive thematic analysis of the interview data to generate themes relevant to our study questions. As a first step, all authors familiarised themselves with the data and identified broad concepts for discussion. VB, RS, and AE then undertook independent line-by-line coding of the data from five separate interviews each (every third interview by study participant number) and identified codes and possible themes. The research team then met to compare possible themes and subthemes and to consider how our personal experience and positioning may be influencing our analysis. This step generated draft themes and satisfied us that sufficient data had been collected to generate themes that would be relevant to our research questions. Using these draft themes as a guide, VB then re-analysed interview transcripts using NVIVO software (Lumivero, LLC. Denver, CO, USA), and identified representative quotes for each theme and subtheme. No new themes were created at this stage. Our author team then met to review this analysis and agreed on the final themes and their precise wording.

Finally, VB reviewed the transcripts to create a list of all faculty development topics mentioned by interview respondents. We felt that this list would be of interest to the translational simulation community, given the heterogeneity of topics we found in the data. All topics were included in the list (Table 1), irrespective of their frequency or importance to the respondents or researchers.

**Table 1:** List of faculty development content topics mentioned by interview respondents

**Knowledge and skills foundational to healthcare simulation**
Scenario design, Curriculum development, Writing learning objectives Technical skills Establishing and maintaining psychological safety, Pre-briefing, Debriefing Program evaluation Simulation-based assessment
**Knowledge and skills specific to translational simulation**
Needs analysis Human factors Return on investment Quality improvement Task analysis SEIPS (Systems Engineering Initiative for Patient Safety) model Safety II Change management Physical design and engineering Design thinking Resilience engineering Process mapping/process improvement FMEA (Failure Mode Effect Analysis) Quality improvement tools -Aim statements and driver diagrams -Root cause analysis / Fishbone diagrams -PDSA (Plan Do Study) cycles Simulation safety System engineering Adverse event management Lean Six Sigma Implementation science

## Results

We sent emails to 18 potential study participants, and 14 agreed to be interviewed. Two additional participants were invited after suggestions from our initial invitees and agreed to participate. Our sixteen participants represented translational simulation programs in Australia, the USA, Scotland, Ireland, Wales, Denmark, Brazil, Norway, and Canada.

We generated three themes in our exploration of translational simulation faculty development practices: (1) *diverse content*, (2) *‘Home grown’, informal processes,* and (*3)* the *influence of organisational context.*

### Diverse content

Overall, the responses relating to the ‘what’ of translational simulation faculty development were less tangible than we expected, and fewer in number than conceptually based literature on translational simulation has suggested [1]. Few programs had a coherent list of learning outcomes for translational simulation faculty development. We have listed topics mentioned by respondents in Table 1 and below we review the subthemes related to knowledge and skills that are (i) foundational to healthcare simulation (ii) specific to translational simulation and (iii) related to stakeholder engagement and change management. A final subtheme—“Unfreezing”—describes the challenges associated with transitioning from the educational to translational mindset.

#### Knowledge and skills foundational to healthcare simulation

Interview respondents felt strongly that there were fundamental skill sets that simulation practitioners required, irrespective of the purpose of the simulation. Most programs offered or required, their staff to be trained in scenario design and delivery and debriefing, and most respondents considered these a precursor to more specific skills for translational simulations. An ability to establish and maintain psychological safety was considered particularly important.

I think the …. core elements of what we do whether it's an education or translational, you know, the importance of psychological safety. Targeting objectives, writing scenarios. So I think that that is the sort of core basics, I think is absolutely vital, [P1]

#### Knowledge and skills specific to translational simulation

Quality improvement and safety science were suggested as important additional areas for skill development for practitioners in translational simulation. Our respondents felt those included both a conceptual appreciation and some practical skills and tools used in these fields.

“Knowledge about some of the commonly applied safety frameworks… thing things like this SEIPS [Systems Engineering Initiative for Patient Safety] model…I think a little bit of safety II, resilience engineering has been applicable and important.” [P5]

“We actually have two full days dedicated to quality within our within our fellowship curriculum. And we're constantly looking for opportunities where we can think about, like return on investment, patient safety, quality improvement, human factors, that type of stuff.” [P7]

Though there was agreement this is important, there was no agreed-upon set of knowledge or skills necessary, but a seemingly endless number that may be useful.

#### Knowledge and skills in stakeholder engagement and change management

An ability to deeply engage with stakeholders was nominated by interview respondents as critical to the success of their translational simulation activities. Social capital was a desired attribute, as were skills in partnering with clinical teams to work on improvement through translational simulation.

“So I think another piece of the faculty development is helping people think about how they effectively engage the right multistakeholder team in these activities in a way that I think has to be much more deliberate than many of our training and performance activities.” [P5]

Given the relatively recent introduction of translational simulation in their health services, advocacy for their programs was also considered important. This included skills in summarising and reporting the findings of translational simulation activities.

“delivering the results or the information gained from those simulations in a in a concise way for the people who hold the purse strings.” [P3]

#### Unfreezing process

Despite a belief in learning ‘simulation basics’ as a foundational element, many of our respondents had experienced a tension; that some of these foundational concepts and practices needed to be adapted or completely re-oriented for translational simulations. They described an *unfreezing* process, similar to that described in Lewin’s three-step model of change [51].

*“*Everyone starts off with the basic strong skill set of simulation - how to create scenarios and how to think about scenario development. But then they there needs to be this whole different... orientation and training. This is a very different scope, this is a very different debriefing process, even the scenarios and the objectives that we create, the materials that we create, and how we prepare participants for them. And the goals are very, very different.” [P8]

Though a commonly described issue, there was little guidance on the content that might help with this transition.

### ‘Home grown’, informal processes

Most programs described ‘homegrown’ approaches to faculty development, with self-directed learning, apprenticeship, and peer mentoring as dominant learning methods. Program leaders lamented a lack of appropriate opportunities in their own career development and felt ill-prepared to lead faculty development for others on their team.

“So really no formal training…very much just kind of looking to…looking to tools from quality and safety, like process mapping, like FMEA [failure modes effect analysis], thinking about how to adapt them for these type of activities, looking at the literature that has come out around that stuff.” [P5]

A smaller number of programs offered structured, formal learning: workshops, seminars, fellowship programs, and/or tiered faculty development pathways. Delivery of these was outsourced in some cases, to harness expertise beyond the translational simulation program.

“Our simulation fellowship program…. run them through an entire year long program where they have 18 Focus days on simulation educator experiences where they do interprofessional work …” [P7]

Many more respondents expressed aspirations and plans to develop more structured offerings.

We have generally had a fairly robust faculty development program for what I would call our training and performance activities. And I think we need to develop analogous courses [for translational sim] [P5]

All respondents highlighted the importance of collaboration and community of practice, both within their institution and across global networks of translational simulation practitioners and scholars.

### The influence of organisational context

The approach taken to faculty development was profoundly influenced by organisational context, particularly the simulation program governance, mission, and strategy.

#### Governance, simulation program mission, and strategy

There were variable governance arrangements for translational simulation programs described by respondents. Some expressed a preference for (or change to) reporting to Quality and Safety, as this enabled more collaboration with relevant teams.

*“Our program is reports to the Chief Quality Officer of our health system, …. And our mission and our aims associated with the hospital operation side are to promote patient safety and quality through innovative education and simulation-based education.”* [P9]

More mature programs had actively organised their work to enable clarity of the purpose of different kinds of simulation (and hence different kinds of faculty development).

“[sim program divided into] ... functional pillars. One of them is what we call our training and performance pillar, which really delivers sort of more traditional education focused simulation, whether that be skills or team training, relational skills, etc. And then we have our human factors and systems design pillar, which is really focused more on translational simulation, looking at hospital processes, environments, quality and safety related *simulation.”* [P5]

#### Collaboration

Collaboration between translational simulation programs and experts in quality improvement, human factors, patient safety, and other domains was frequently mentioned in interviews. This collaboration was for both successful translational simulation practice, but also to support faculty development (informal or formal) for simulation practitioners.

“They went through a QI quality and safety curriculum that was offered, thankfully, outside of our sim program, because we didn't have the bandwidth to do such but we partnered with our quality and safety departments.” [P8]

“We from simulation, we have pushed ourselves and we’ve invited ourselves into quality improvement. We’ve knocked on their doors. We’ve asked them to be involved. We’ve tried to encourage people who know quality improvement to learn about simulation, or people who know about simulation to learn about quality improvement, or collaborate with their unit quality improvement.” [P6]

Whether by accident or design, translational simulation programs variably emphasised three approaches: developing faculty knowledge and skills, developing tools to support faculty, or having a diverse faculty (a balance of recruiting versus developing). Many programs recognised their lack of expertise in the areas of QI or patient safety and sought instead to develop tools that supported a consistent approach to practice.

*“*And so we're trying to be a bit more prescriptive and systematic, we're like using Kerns [curriculum framework], but then using things like aim statements, and like Fishbone diagrams and things like that, to help support our staff.” [P7]

“Now, internally we have a facilitator guide template, a debriefing template and FMEA [failure mode effects analysis] scoring tool and an FMEA scoring report, like template to generate the report.” [P13]

## Discussion

Leaders in translational simulation programs consider a diverse array of knowledge and skills important for translational simulation faculty and report mainly informal approaches to the development of these skills. In reflecting on our findings, we are struck by the influence of institutional context and collaboration in shaping faculty development approaches. These findings are consistent with our experience and included even more diverse content than we anticipated. In this discussion, we consider the implications of our findings for leaders, practitioners, and scholars working in translational simulation practice.

In examining ‘faculty development’ within the translational simulation community, we came to reflect on a broader question; how do translational simulation programs *build capacity*? Capacity is much more than a shopping list of individual skill sets. It requires clear program objectives, effective governance and operational processes to support the achievement of those aims, and appropriately skilled simulation practitioners. Given the breadth of knowledge and skills potentially required for translational simulation activities, programs may be well served to make intentional choices about recruitment of, and collaboration with, individuals and teams with complementary expertise.

Our findings illustrate that the choices translational simulation programs make will be informed by institutional context, including alignment with service priorities. If a new hospital is being built, the translational simulation program will require expertise in human factors, design thinking, and change management, and will require collaboration with experts in these fields as well as architects and capital works teams. If a strategic aim is a responsive simulation service to address emerging quality issues in a health service, the simulation practitioners will require expertise in quality improvement methods and techniques and stakeholder engagement. If translational simulation is viewed as a strategy for shaping culture in a health service, the faculty team will need to draw upon perspectives from anthropology and organisational psychology.

Given the importance of this institutional context, we were not surprised by the lack of formal courses offered to individual simulation practitioners. Although the *‘homegrown’* descriptor offered by our respondents might suggest ad hoc or immature approaches, we prefer to consider this appropriate adaptability in emerging contexts. There is a tension between training in general principles, which may be too broad to be practically helpful, and having granular toolkits which may not be relevant in every context. This tension is relevant to simulation faculty development more broadly [24], but is of particularly importance for translational simulation programs.

That said, we found some commonalities in responses from translational simulation program leaders. Foundational knowledge and skills in simulation practice were important, e.g. scenario design, technical delivery, leading learning conversations, participant engagement, and psychological safety. However, there were specific needs for translational simulation. The dilemma of ‘unfreezing’ was a common finding, and perhaps reflects the strong educational paradigms underpinning most simulation practitioners’ experience prior to their involvement in translational simulation. Quality improvement and safety science expertise were identified as important, including methods and tools drawn from these fields. This finding aligns with numerous published examples of translational simulation projects using ‘QI’ tools [2, 35, 36].

Our work has limitations. While we suggest that findings are informative, we do not claim they are a comprehensive picture of faculty development for translational simulation. We are also aware the perspectives of the program leaders we interviewed may not be those of the simulation practitioners and faculty who are the subject of the formal and informal faculty development strategies described. Our findings are interpreted through the lens of our own conceptualisations of translational simulation and experiences of faculty development. We have attempted to attend to reflexivity throughout data collection and analysis by searching deliberately in the data for experiences and opinions that were different than our own but recognise the limitations in doing so.

Our messages for *translational simulation program leaders* seeking to develop faculty development strategies are threefold. Firstly, translational simulation programs are in good company with ‘homegrown’ approaches that are highly contextually bound. Secondly, that clear articulation of program aims, strategies and scope will be helpful in developing and articulating a coherent faculty development approach that is institutionally focused. Thirdly, that collaboration will serve programs well in building capacity for a broad range of translational simulation targets, including, but not limited to, skills and expertise. These messages align with calls to critically re-evaluate the benefit of “isolated continuing education offerings, e.g. workshops” for individual health professional educators [30].

Our lessons for *translational simulation practitioners* are to seek clarity about the aims and scope of the translational simulation programs in which they are employed and to consider internal and external opportunities to gain relevant knowledge and skills as well as collaboration.

Our lesson *for researchers and scholars* working in simulation faculty development—for translational and educationally focused programs—is to go beyond *content* and *process* issues for training individual practitioners, and to examine the influence of context and collaboration in building capacity for simulation delivery. This is not to discredit attempts for consensus or consistency in learning objectives for translational simulation faculty development. Nor is it to refute evidence-informed practices for training or credentialing frameworks. Rather, to celebrate nuanced approaches that build on fundamental knowledge and skills, tailored to the aims and strategy of institutional simulation programs.

## Conclusion

Faculty development for translational simulation is embryonic, with even established programs taking predominantly informal approaches. There is a need for practitioners to develop knowledge and skills from diverse fields such as quality improvement and safety science, but the content should be informed by program aims, scope, and strategy. Collaboration beyond the historical boundaries of the healthcare simulation community is valuable, both for developing practitioners’ skills and for building an effective practitioner team within translational simulation programs.

### Supplementary Information

**Additional file 1. **Participant Information and Consent Form (PICF).

**Additional file 2. **Interview guide

## Data Availability

The datasets generated and/or analysed during the current study are not publicly available to protect the confidentiality of interview participants. Deidentified data may be available from the corresponding author on reasonable request.

## Abbreviations

SBE: Simulation-based education
SRQR: Standards for Reporting Qualitative Research
SESAM: Society for Simulation in Europe
SEIPS: Systems Engineering Initiative for Patient Safety
FMEA: Failure Modes Effect Analysis
QI: Quality improvement
