# Supplementary material for: Faculty development for translational simulation: a qualitative study of current practice
Source: Adv Simul (Lond). 2023 Nov 2;8:25. doi: 10.1186/s41077-023-00265-0 (PMC10621189; doi:10.1186/s41077-023-00265-0)
Supplement: Supplementary file 1 — Additional file 1. Participant Information and Consent Form (PICF). [file 41077_2023_265_MOESM1_ESM.pdf]

Consent Form

Project: Faculty development for Translational Simulation: a review of current practice
Chief Investigator: Victoria Brazil

1. What is the research study about?

Recent enthusiasm for translational simulation in healthcare has not been supported by clear guidance for training the practitioners who deliver these simulations.
If translational (or '*systems/ QI focused'*) simulation purports to directly improve quality in healthcare, then faculty need additional skills; building on those required for educationally focused simulations. Tools and perspectives from the fields of quality improvement, implementation science, and change management are relevant, but rarely featured in simulation faculty development programs.
Despite a lack of published, evidence-based practice, we are aware that translational simulation programs around the world are supporting their faculty to develop these skills. We seek to explore and describe these practices – the objectives, pedagogies and methods – in how faculty are prepared for the design, delivery and debriefing of translational simulation activities.
With an informed picture of current faculty development strategies and approaches, our translational simulation community can move toward a consistent approach, and shared expertise.

2. Who is conducting this research?

This research is being carried out by Victoria Brazil, Rebecca Szabo, Alexander El Kheir and Eve Purdy, who are experienced in healthcare simulation and associated research and scholarship.

3. Can anyone take part in this study?

We are looking to recruit people who have leadership and/or faculty development responsibilities within translational or systems-focused simulation programs in healthcare. This may include but is not limited to :-

4. What does the participation in this study involve?

Our aim is to explore current faculty development practices within translational simulation programs, rationale for the choices made, and perceived effectiveness for delivery of translational simulation activities.

You will participate in an interview with one of the research team that explores current approaches to faculty development for this type of simulation. This interview will occur via Zoom at time convenient for you and last between 45-60 minutes. With your permission the research team would like to audio record the interview, and it will subsequently be transcribed and de-identified.

You will be sent the transcript within one week after completion of the interview, and may withdraw up to two weeks after this. During that two week period you are also invited to provide any comments or validation of the content. After that two week period, the de-identified transcript will be entered into data analysis and you will not be able to withdraw the data from the study.

If you are unable to participate in an interview you can complete a 20-30 minute online questionnaire through Qualtrics. This will be completed anonymously and so there is no opportunity to withdraw your participation after submission.

5. Will I incur any costs by participating in the study?

There are no costs associated with participating in this research project, nor will you be paid.

6. Will the study involve any risk or discomfort?

We anticipate the risk of minor inconvenience only.

Although it is unlikely, you may feel that some of the questions we ask are stressful or upsetting. If you do not wish to answer a question, you may skip it and go to the next question, or you may stop immediately. If this occurs during the interview, the researcher will provide details of support services relevant to your country/ location, or may encourage you to access employee assistance schemes provided by your employer.

7. What will happen to the information collected about me?

By clicking “yes” you consent to the research team collecting and using information provided by you for the research study.

The research team will store the data collected in a secured location at Bond University for:

- 5 years after the publication of the research results

The information about you will be stored in a:

• Non-identifiable format where your identify will be unknown

8. What if I want to withdraw from the research study?

Participation in this study is voluntary, you are not under any obligation to consent and if you do consent you can withdraw at any stage up to 2 weeks after being sent your interview transcript. This will not affect your relationship with Bond University, or any other organisations involved in this research.

You can withdraw your consent by advising the researcher verbally, via email or by completing and returning the “Participant Withdrawal of Consent Form” that is supplied as part of this information pack.

You may stop the interview at any time if you do not wish to continue. The data and audio recording will be erased and will not be included in the study.

9. How do I obtain further information about this study?

If you would like to know more information regarding this study, or if you have any problems which may be related to your involvement in this study you can contact the research team:

**Name**	Victoria Brazil
**Position**	Director, Translational Simulation Research Collaborative
**Telephone**	(+61) 0418 981 538
**Email**	vbrazil@bond.edu.au
**Ethics Approval Number**	VB00057

10. Will I receive the results of the Study?

The research team intend to publish and/ report the results of the research. If you would like a copy of the results please indicate this on the consent form.

11. What if I have a complaint or any concerns about the research Study?

Any concerns or complaints about the conduct of this study should be direct to:

Bond University - Human Research Ethics Committee
14 University Drive
ROBINA QLD 4226
Email: **ethics@bond.edu.au**
Phone: +61 7 5595 4194

This information sheet is for you to keep.

Participant providing consent

- I understand I am being asked to provide consent to participate in this research study;
- I have read the Participant Information Sheet, or someone has read it to me in a language that I understand;
- I understand the purposes, study tasks and risks of the research described in the study;
- Recordings: I understand that the research team will audio/video record the interview; I agree to be recorded for this purpose.
- I provide my consent for the information collected about me to be used for the purpose of this research study and for any future research
- I have had an opportunity to ask questions and I am satisfied with the answers I have received;
- I freely agree to participate in this research study as described and understand that I am free to withdraw from the study and withdrawal will not affect my relationship with any of the named organisations and/or research team members;

I agree to participate in this study ☐ **Yes** ☐ **No**

At the completion of the study would you like a copy of the results ☐ **Yes** ☐ **No**

Withdrawal Form

Project: Faculty development for Translational Simulation: a review of current practice
Chief Investigator: Victoria Brazil

Withdrawal of Participation

You can withdraw your participation consent by advising the researcher verbally, via email to vbrazil@bond.edu.au or by returning this completed form.

I wish to WITHDRAW my consent to participate in this research study described above and understand that such withdrawal WILL NOT affect my relationship with Bond University, other participating organisation[s] or other professional(s).

Participant Name

Name of Participant or identification number (please print)	
Participant signature	
Address	
Email	
Phone	
Date	

The section for Withdrawal of Participation should be forwarded to:

Chief Investigator Name:	Victoria Brazil
Email:	vbrazil@bond.edu.au
Phone:	(+61) 0418981538
Postal Address:	Bond University<br>14 University Drive,<br>Robina, QLD 4226
