# Supplementary material for: Faculty development for translational simulation: a qualitative study of current practice
Source: Adv Simul (Lond). 2023 Nov 2;8:25. doi: 10.1186/s41077-023-00265-0 (PMC10621189; doi:10.1186/s41077-023-00265-0)
Supplement: Supplementary file 2 — Additional file 2. Interview guide [file 41077_2023_265_MOESM2_ESM.docx]

**Faculty Development for Translational Simulation Study**

**INTERVIEW GUIDE**

*Thank you for agreeing to participate. We anticipate this interview will take about 45-60 minutes. It will be recorded then transcribed using a digital transcription service (e.g. OtterAI). We will de-identify the transcript my removing your name, the name of anyone you mention, and the names any institutions you mention. Any further information that is identifiable based on specific context will not be included directly in the presentation of results but may inform our analysis. We will email you the de-identified transcript within a week of completing the interview.*

*During this interview, we will pose questions about your simulation program, with a particular emphasis on translational or systems focused simulation, and on faculty development for those designing and delivering these simulation activities.*

*What questions do you have? May we proceed?*

1. Tell us about the simulation program you are involved in.
   - Aims and objectives
   - Scope and institutional context ( i.e. what kind of hospital or health service partnerships), country
   - Staffing and governance
   - History/ years of operation
   - Faculty development workshops or programs
2. What is your role(s) in the program?
3. How much of your simulation program is ‘translational’ or ‘systems/ QI focused’?
   - What does that involve? Can you give examples?
   - What is the aim of doing this kind of simulation work in your context?
   - How is the planning or strategy set for your translational simulation activities?
4. Tell us about the faculty/ facilitators / staff that design and deliver these translational simulation activities
   - What is their background, training, recruitment?
   - Are they the same team that deliver educationally focused simulations in your program?
5. What knowledge, skills or perspectives do you think are required for the successful design and delivery of your translational simulations?
   - *[Prompts] …… Technical, scenario design, QI tools, patient safety tools, debriefing skills, leadership/ change management, other?*
   - How do you think these are the same as, or different to, knowledge and skills for educationally focused simulations?
6. How do your staff currently acquire those knowledge/ skills/ perspectives for translational simulation? What kind of faculty development occurs?
   - **Tell me about the experience of a new person who arrives and wants to do ‘translational sim’?**
   - Why have you/ your program chosen that approach?
   - What resources do have available to support this work?
   - Are there external courses or programs you can access?
   - How do you evaluate your faculty development efforts?
7. What else would you like to do that builds faculty capacity for design and delivery of translational simulation?
   - What are the barriers to that?
   - What is your ‘dream faculty development’
   - How to recruit for the mix of faculty required for faculty
8. Anything else you’d like to add?
9. Anyone else we should talk to?

*Many thanks for participating*.
